# The Role of Frustration in Human–Robot Interaction – What Is Needed for a Successful Collaboration?

**DOI:** 10.3389/fpsyg.2021.640186

**Published:** 2021-03-22

**Authors:** Alexandra Weidemann, Nele Rußwinkel

**Affiliations:** ^1^Cognitive Modeling in Dynamic Human-Machine Systems, Faculty of Mechanical Engineering and Transport Systems, Department of Psychology and Ergonomics, Technical University of Berlin, Berlin, Germany; ^2^Junior Research Group MTI-engAge, Control Systems Group, Department of Electrical Engineering and Computer Science, Faculty of Electrical Engineering and Computer Science, Technical University of Berlin, Berlin, Germany

**Keywords:** human–robot interaction (HRI), frustration, collaboration, influence, recommendations

## Abstract

To realize a successful and collaborative interaction between human and robots remains a big challenge. Emotional reactions of the user provide crucial information for a successful interaction. These reactions carry key factors to prevent errors and fatal bidirectional misunderstanding. In cases where human–machine interaction does not proceed as expected, negative emotions, like frustration, can arise. Therefore, it is important to identify frustration in a human–machine interaction and to investigate its impact on other influencing factors such as dominance, sense of control and task performance. This paper presents a study that investigates a close cooperative work situation between human and robot, and explore the influence frustration has on the interaction. The task for the participants was to hand over colored balls to two different robot systems (an anthropomorphic robot and a robotic arm). The robot systems had to throw the balls into appropriate baskets. The coordination between human and robot was controlled by various gestures and words by means of trial and error. Participants were divided into two groups, a frustration- (FRUST) and a no frustration- (NOFRUST) group. Frustration was induced by the behavior of the robotic systems which made errors during the ball handover. Subjective and objective methods were used. The sample size of participants was *N* = 30 and the study was conducted in a between-subject design. Results show clear differences in perceived frustration in the two condition groups and different behavioral interactions were shown by the participants. Furthermore, frustration has a negative influence on interaction factors such as dominance and sense of control. The study provides important information concerning the influence of frustration on human–robot interaction (HRI) for the requirements of a successful, natural, and social HRI. The results (qualitative and quantitative) are discussed in favor of how a successful und effortless interaction between human and robot can be realized and what relevant factors, like appearance of the robot and influence of frustration on sense of control, have to be regarded.

## Introduction

Robots are no longer just tools in industrial context. Soon, robots will become part of our daily life. The vision is that robots interact with humans in close collaboration without security shelters in between. In a collaborative situation, according to Onnasch et al. (2016) humans and robots work on common goals and subgoals, which are assigned according to the situation during the collaboration and take place in the same workspace.

The challenge for human–robot interaction (HRI) research is to design a successful and enjoyable interaction. The identification and measurement of factors that play a relevant role in successful collaborations is crucial regarding the design and development of a suitable robot system and the direct interaction. If the robot does not meet the requirements, needs and perspectives of the user, or if those are not taken into account, the robot will most probably not be accepted by the user (Davis, 1989; Venkatesh et al., 2003; Heerink et al., 2007; Broadbent et al., 2012; Smarr et al., 2014). Various lines of research (such as Riek et al., 2009; Waytz et al., 2010; Salem et al., 2015; Abd et al., 2017; Ciardo et al., 2018; Onnasch and Roesler, 2019) investigated different aspects like trust, appearance, anthropomorphism, and acceptance that play a role in HRI. An important aspect of human-centered research in HRI are human emotions during the interaction, especially negative emotions. One negative emotion that is often mentioned in dealing with technology, is frustration (Ceaparu et al., 2004; Lazar et al., 2006). Frustration arises when a person has the expectation to reach a goal but still fails to achieve it after repetitive attempts (based on Freud, 1921; Russell, 1980; Amsel, 1992; Scherer, 2005; Bortz and Doering, 2013).

### Expectations

Humans have specific expectations regarding the details of the interaction with a robotic system based on, e.g., the appearance of the robot system, the way of conducting the task with the system often relating to the similarity to human–human interaction (HHI), like the way of communication (verbal and non-verbal) and social behavior toward the interaction partner and social norms (Compagna et al., 2016; Beer et al., 2017; Jerčić et al., 2018). Humans use HHI mechanisms, like proxemic behavior, interpretation of the other’s intention, the way of communication, and social, physical, behavioral cues, to perceive robots as autonomous social agents, as socially present human employees (Fiore et al., 2013). It has been shown that humans treat computers as teammates with personality (Nass et al., 1995, 1996). Humans tend to behave socially not only toward other humans but also toward robots (Reeves and Nass, 1998; Dautenhahn, 2007). Without prior training, humans prefer natural and intuitive communication in use with the technical system (Dautenhahn et al., 2005). There is a tendency for people to prefer human-like attributes in robots (Kiesler and Hinds, 2004; Walters et al., 2008). It has been shown that humans were better able to empathize with this type of robot (Riek et al., 2009) and this assumingly leads people to ascribe human-like mental abilities to the robot (e.g., intentions, emotions, cognition) (Waytz et al., 2010; Schneider, 2011).

Regarding the question on how to realize successful collaborative working situations, it is helpful to analyze human–human collaboration situation. Humans have developed a number of abilities to achieve joint action (Sebanz et al., 2006). Mechanisms such as joint attention and other cognitive mechanisms for sharing representations of objects and events as well as common task knowledge help us to initiate and coordinate joint action. Whenever actions of the partner indicate a mismatch of the representation of the common goal and the way of how to achieve this goal, an immediate facial expression follows and informs the partner without too much explicit communication. Therefore, such emotional facial reactions could also be a very relevant indicator for a successful human-robot collaboration.

To evaluate human reactions to different kinds of robots with varying outer appearance, many studies have used pictures or videos (e.g., Bartneck et al., 2007). However, two-dimensional images cannot represent the complex three-dimensional appearance, movement and sounds of social HRI (e.g., Wainer et al., 2007). Therefore, it is important for studies investigating HRI, to use at least two different kind of robots (with differences in human-like appearance) to prevent a misinterpretation of behavior and considering a broader variability of reactions to different robotic systems.

For these reasons it is interesting to consider the appearance of the robot, expectations that arise and to draw comparisons to HHI for designing robot systems and HRI.

### Negative Emotion – Frustration

If the expectations of a human partner on the robot are disappointed or not fulfilled, negative emotions like frustration can arise and even lead to the termination of the interaction. Emotions can occur during all kind of actions and mental operations (Picard, 1997), they motivate actions and have influence on performance, trust, and acceptance during an interaction and on the interaction behavior itself (Brave and Nass, 2002).

The emotional experience of frustration can be caused by simple events such as time delays and errors that can occur due to lack of knowledge and insufficient training in human–computer interaction (HCI) (Bessière et al., 2004; Lazar et al., 2006). Working with a computer agent that the user does not trust leads to the development of frustration (Hirshfield et al., 2011). Examples in the literature of frustration in HRIs are situations such as e.g., behavioral errors by the robot like dropping a bottle or moving to the wrong takeover-location in a bottle handover task with the robot (Abd et al., 2017). In interactive situations with different robots, the participants are more frustrated by such kinds of technical failures than when experiencing a social norm violation, for example “not looking directly at the person it is talking to” (Giuliani et al., 2015, p. 3) (Giuliani et al., 2015). For these reasons, technical failures were used in our study (see also section “Experimental Description”). Such technical failures could be used in studies to intentionally induce frustration to participants in such interaction situations to generate a perceived increase in frustration. In such cases humans usually show immediate emotional feedback to the robot in form of reactions such as facial expressions (Lang et al., 2010).

Frustration leads to lower task productivity (Waterhouse and Child, 1953; Klein et al., 2002; Powers et al., 2011), slower response times (Chen et al., 1981), longer decision-making time (Lerner et al., 2015), prolonging content acquisition on learning (Amsel, 1992), and lower learning efficiency (Kort et al., 2001; Graesser et al., 2005; Woolf et al., 2009). Decreased motivation (Weiner, 1985), user satisfaction, and lacking trust (Lazar et al., 2006; Hirshfield et al., 2011) are evoked by frustration. It was found that frustration triggers a rise in arousal, which enhances cognitive performance, and is associated to high workload (e.g., Whinghter et al., 2008). Therefore, whenever the perception of frustration could be prevented, this would cause a benefit on the further interactive process and the quality of the task conductance.

Various authors found a direct influence on the acceptance of a technical system and trust on the decrease of frustration (Giuliani et al., 2015; Yang, 2016; Abd et al., 2017). It was found that the sense of dominance was low when frustration was high in a task with high attentional demands (Weidemann and Rußwinkel, 2019). In this study, dominance was viewed and questioned as control and the ability of being in control of a situation. The concepts of dominance and control in the study described in this paper were considered separately by extending the SAM questionnaire (for more details see section “Questionnaires”). The dominance dimension in the SAM questionnaire represents changes in degree of control, the maximum control in the situation is presented by a large figure (Bradley and Lang, 1994). In this study, “dominance” is defined as superiority in interaction and also over the interaction partner and “control” as control in the situation, over one’s own action and through action, i.e., also as the difference between the perception of an event in a situation and the intended effect (Pacherie, 2007; Haggard and Chambon, 2012). Dominance is an important factor for the judgment of the interaction, partner and communication in a social interaction (Ng and Bradac, 1993; Berger, 1994). The importance of dominance has also been shown in the results of the SAM questionnaire in our past study on frustration.

The two terms sense of control and sense of agency are connected in psychology. Sense of agency refers to “being in control both of one’s own actions and through them” (Haggard and Tsakiris, 2009, p. 242). Being able to realize intended actions and the expected outcome with the robot would therefore result in a higher sense of control. Such a factor is interesting in regard to how successful a tool is used for a certain aim as well as how successful I am in an interaction with another person e.g., “am I successful in order to make myself understood by the other person,” or in other words, “do I experience the intended effect that I tried to cause by my actions?” Ciardo et al. (2018) suggest that sense of agency is negatively affected by frustration in the interaction with an embodied robot similarly, to interacting with other humans.

In that sense repeated unsuccessful HRIs related to a chosen aim leads to perceived frustration of the human partner. The identification of such unsuccessful frustrating events would enable the implementation of solution functions, e.g., for the HHI.

As can be seen, it is important to be able to identify and minimize frustration. Emotions are object-directed and have a characteristic experience and the occurrence of physiological changes and behavioral patterns is evident (Klug, 2012). In the literature several methods are reported to access emotions, these can be divided into subjective (like questionnaires) and objective (like psychophysiological methods) methods.

### How to Measure Emotions

Subjective measures of emotions such as self-report methods are efficient and easy to administer, they are beneficial to determine emotions. However, participants are susceptible to time effects and may respond based on social desirability (Mauss and Robinson, 2009; Lopatovska and Arapakis, 2011) or have no direct access to the emotional experience.

During the experience of emotions specific physiological changes occur in the human body (Peterson et al., 2015). Because the measurements of such physiological changes can be recorded parallel to the occurrence of the emotion in contrast to subjective methods. An additional use of psychophysiological methods would support the determination of emotions. Vyzas and Picard have shown correlation between various emotions (such as joy and frustration) and physiological signals (like pulse and galvanic skin response) (Vyzas and Picard, 1999). On the downside, physiological measurements are ambiguous, and the best methodological combination of measurements remains to be found especially regarding different experimental settings.

The multicomponent phenomenon frustration often occurs during human–machine interaction (Ceaparu et al., 2004; Lazar et al., 2006) and initiates not only changes in facial expression, but also in posture, physiology, or behavior (Scherer, 2005). It was found that heart rate variations are sensitive to frustration and the heart rate itself is positively correlated with this emotion (Wulfert et al., 2005; Washington and Adviser-Jones, 2011; Yuan et al., 2014). During incorrectly completed tasks, facial muscle activity may also provide evidence of frustration (Jost, 1941; Hamm et al., 2011; Hazlett, 2013; Gao et al., 2014; Lerner et al., 2015). But all these findings are not robust enough to be used in isolation to measure frustration. Therefore, a multi-method approach to measuring frustration is used in this study.

### Aim

It seems that the emotional experience of frustration and its influence on interaction factors, and interaction quality could provide a good guideline for the evaluation of robot systems, and for the recommendation of the design of a pleasant and successful HRI.

To gain a deeper understanding of these possibilities, we follow one main question in this paper:

How does frustration influence HRIs?

To investigate this question a human–robot collaborative experiment was designed, consisting of a task with a common goal including handover scenarios. The participants interacted with two different robot systems, investigate the range of changes in behavior due to the technical system used. In the experiment different measurements of frustration were applied, which have been used before in similar studies. One aim of the study was to induce and measure frustration, among others with questionnaires. The second aim was to investigate the influence frustration has on the HRI.

### Hypotheses

Based on findings from related work on frustration and robot appearance in psychology, HCI and HRI, we developed four hypotheses for the study:

H1: Technical errors by the robot lead to perceived frustration by the participants.
H2: Frustration leads to decreased dominance, sense of control, and self-reported performance.
H3: Frustration leads to lower rating regarding acceptance of the robot systems.
H4: The interaction with the more human-like robot (here “*Pepper*”) is preferred, among other aspects due to the human-like appearance and similarity to HHI. This leads to an attribution of human-like abilities to the robot and to a tendency to forgive mistakes, in contrast to a more technical looking robot that would be expected to behave more precise.

## Materials and Methods

### Experimental Motivation

A collaboration task was chosen to investigate direct interactions, since the human shares the workspace with the robot to perform the common task. In this study the interaction corresponds to a task processing (in the following called interaction task).

A good example of a close interaction task is a handover scenario with a robotic system. Since different colored balls needed to be handed over from the human to the robot and be placed in specific baskets relevant components such as feedback (robot and human), joint actions and giving and perceiving instructions were relevant for the quality of task completion. Similar scenarios have been investigated elsewhere (Rasmussen, 1982; Giuliani et al., 2015; Abd et al., 2017; Honig and Oron-Gilad, 2018) with differing research questions. For the scenario in the present example, technical execution failures of the robot were initiated, like dropping the ball, to induce frustration.

A task with a common goal is helpful for the development of negative emotions, such as frustration. After all, not achieving a common goal that is relevant to you because your partner fails can lead to frustration.

**FIGURE 1:**
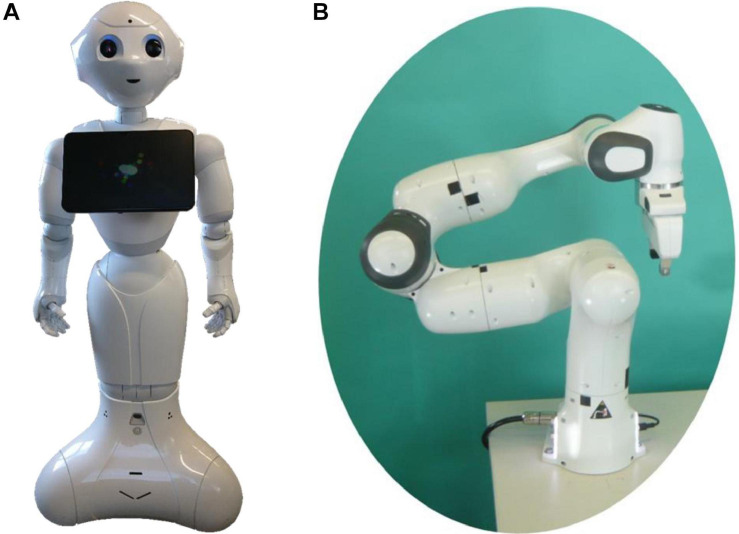
**(A)** On the left the robot “*Pepper*” from Aldebaran Robotics SAS and SoftBank Mobile Corp and **(B)** On the right the robot “*Panda*” from FRANKA EMIKA. The photos of the robots were taken and edited by Alexandra Weidemann.

Two different robot systems (Figures 1A,B) were used taking into consideration that the appearance, movement could form different expectations and might have a strong influence on the interactive behavior of the participant and on the evaluation of the interaction task. For a systematic investigation of such kind of influences a broader variety of robotic systems would have been necessary. In other studies, usually only one type of robot is investigated. In the study described here, a person is working on the same task interacts with two systems (one after the other), so they can (be) compared directly. The requirements of the two to be chosen robotic systems were (1) the ability to physically interact with the participant (at a similar paste) and (2) to find two systems that differ in humanoid appearance, such as a social and industrial robot (Chanseau, 2019).

The methods (questionnaires and interviews) used have already proven in other studies to determine emotions or even frustration. In addition, these methods have been investigated based on a multimodal approach in order to investigate which methods are best suited to measure frustration in HRIs.

Questionnaires, video recordings (to counterbalance the self-assessment problem (Bethel and Murphy, 2010) and to evaluate reactive behavior showed by participants) and interviews (to provide further insights into the participants state of mind) are frequently applied as methods in the observation of interactions in various studies and were also used here (Chanseau, 2019).

Feedback given by the robot, in our case status of the system (open for instructions or not), is very important for good communication between two parties in an interaction. Here the chosen feedback channel was visual and realized as LED-feedback, which has been shown to be useful for example in a study by MTI-engAge project.

### Experimental Description

#### Study Design and Participants

The HRI study was done in a between-subject design with 30 healthy participants [age: 18–35 years; *N* (male) = 14, *N* (female) = 16]. The average age was 29.1 (*SD* = 5.2). Subjects were recruited via notices at universities in Berlin and the subject portal of the Technical University of Berlin. The subjects were randomly divided into two condition groups: frustration (FRUST) and no frustration (NOFRUST) which was considered as independent variable.

#### Technical Systems

##### Robotic systems

The subjects interacted with two different robot systems, a humanoid robot (“*Pepper*” from Aldebaran Robotics SAS and SoftBank Mobile Corp) and a robotic arm (“*Panda*” from FRANKA EMIKA). The robots were controlled by a Wizard-of-Oz scenario (controlled by a specially written computer program), so the experimenter generated the reactions of the robots during the interaction tasks for practical and safety reasons.

##### LED-feedback

To enable the robot to give feedback in response to a “trigger input” from the subject an LED-feedback was developed. The robots gave feedback to the human about their current “state” via three colors of a LED lamp. If the LED was “green,” instructions (with the help of gestures and/or words) could be given to the robot. If the LED was “orange,” the robot “processed” the input from human. If the LED turned “red,” then the robot either did not understand the input or the input was incorrect.

#### Experimental Setup

**FIGURE 2:**
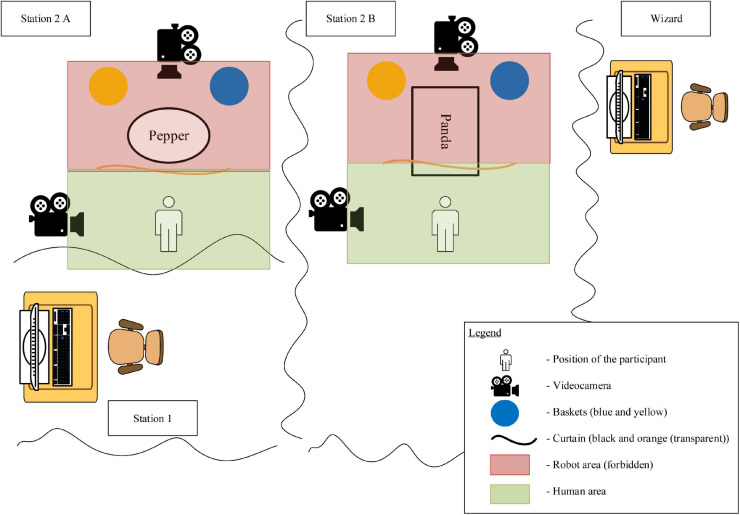
Setup of the human–robot interaction experiment from above.

The interaction tasks (one with “*Pepper*” and one with “*Panda*”) took place in rooms separated by curtains, so that the subjects were “alone” with the robot and visually shielded from other people (see Figure 2). Each interaction-place was divided into two areas: the area for the human (green area) and the area of the robot (red area), which the human was not allowed to enter with any part of the body. The subject changed stations during the experiment. At station 1, the subject filled out the questionnaires before and after the interaction tasks. The interaction tasks took place at station 2. The Wizard of Oz’s (the person that controlled the robot) seat was at robot height and hidden behind the curtains. From there, the wizard was able to observe the participants with the help of cameras above the station 2, and controlled the robot.

#### Experimental Procedure

**FIGURE 3:**
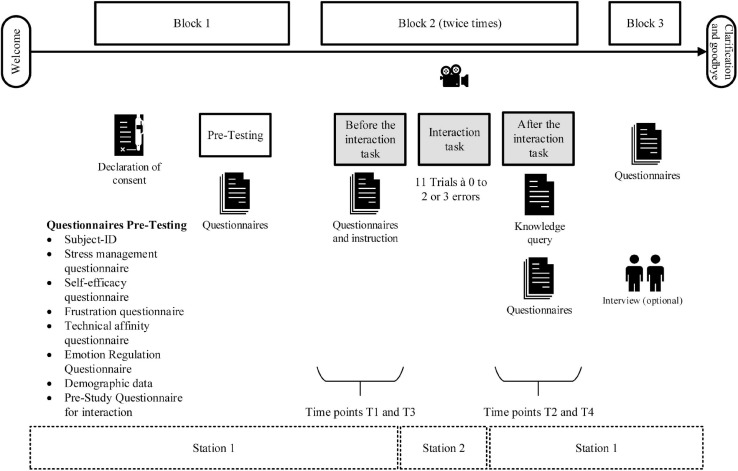
Procedure of the human–robot interaction experiment.

The procedure of the experiment was divided into three blocks (Figure 3). In the first block, general questionnaires (pre-testing) were filled out. The interaction tasks with a robot system (at station 2) and the completion of the corresponding questionnaires took place in block 2. Thus, the participants performed block 2 twice. The interaction tasks with the two robot systems took place successively in randomized order. This served to avoid a sequence effect. Questionnaires about the health of the human interaction partner, the knowledge about the triggers, the robot system, and the interaction were given at four different time points throughout the block 2, before each interaction task (T1 and T3) and after each interaction task (T2 and T4) (at station 1). In block 3 final questionnaires regarding both interaction tasks were filled out and optionally an interview was performed.

Each interaction task in block 2 included 11 trials, since a maximum of 11 balls should be handed over (handover scenarios). Within a trial, no errors or two to three errors could occur. In the FRUST-group, errors occurred in nine trials (trial 2, 3, 4, 5, 6, 8, 9, 10, and 11). In 2 trials (trial 4 and 7) errors occurred in the NOFRUST-group. The experimenter determined, according to this rules, in which trial errors arose before the study started.

##### Block 2: interaction task

The different handover situations with the robots were controlled by a Wizard-of-Oz scenario (controlled by a specially written computer program and the experimenter). These are handover scenarios in which the subject should give colored balls (yellow and blue) to the robot and, with the help of gestures and words, get the robot to throw the ball in a corresponding colored basket in the room separated from the human.

The participants had two subtasks. In the first subtask, at least three balls of each color had to be placed with the help of the robot into the corresponding basket. In the second subtask the participants had to find out which gestures and words, so-called triggers, caused the robot to react and release the ball into the basket. The type of trigger words (color, direction) and trigger gestures (pointing gesture, color card) were known by the participants, but not which robot reacted to which corresponding trigger (word or gesture) or trigger combination (word and gesture) with the desired reaction (release of the ball into the corresponding basket). The participants stated their knowledge about the triggers in the knowledge inquiry at the end of the experiment (see also section “Questionnaires”).

The interaction tasks were divided into four different phases:

(1) attracting
(2) handing over the ball
(3) choosing the trigger
(4) the robot’s passing of the ball.

In the attracting phase, the subjects should attract the robot, for example by calling over, so it would moves toward the human to receive the ball with the robot’s gripper for the ball transfer. After the ball was successfully handed over, the robot moved to a so-called “waiting position” and the subject could select the trigger to find out how the robot reacts to the trigger. This was also supported by the LED-feedback. If the trigger was selected correctly, the robot released the ball into the corresponding basket in its area.

The technical errors caused by the robot occurred during the ball handover phase of the four interaction task phases. There were four different types of technical errors:

(1) the gripper remained open
(2) the gripper remained closed
(3) the gripper picked up the ball and dropped it in the area of the human
(4) the gripper picked up the ball and dropped it in its area.

There were more errors in the FRUST-group than in the NOFRUST-group, so the subjects in the FRUST-group were supposed to experience frustration.

During the interaction tasks, video recordings (from the front and from the side, see also Figure 2) were made. Short interviews were conducted with a certain number of subjects about the interactions.

#### Questionnaires

All questionnaires were filled out on the computer.

The pre-testing phase in block 1 included questionnaires on the affinity for technology, general well-being, and emotion regulation.

The following described questionnaires expect the post-post study questionnaire were given at four different points in time throughout the experiment, before each interaction task (T1 and T3) and after each interaction task (T2 and T4).

The three following questionnaires have to be filled out before (T1 and T3) and after the interaction task (T2 and T4) (Figure 3, see block 2). A 6-scale questionnaire about different emotions (like satisfaction and frustration) and condition (like tiredness) of the human (EaCQ) was based on Positive and Negative Affect Schedule (PANAS) (Watson et al., 1988; Krohne et al., 1996) and BSKE21 (Janke et al., 1988, 1995; Janke and Debus, 2003). This questionnaire and the self-assessment manikin (SAM) (Bradley and Lang, 1994) ranged from 1 to 6. SAM and EaCQ were performed to be able to evaluate the emotional state over the task period. The third questionnaire was the NASA‘s Task Load Index (NASA- TLX) (Hart and Staveland, 1988), which was used to determine task performance and frustration. The scale was converted linearly into percentage scales. These questionnaires were already used in other literature to identify changes in emotions, especially frustration (for example Yuan et al., 2014; Ihme et al., 2016, 2017, 2018).

The SAM questionnaire (Bradley and Lang, 1994) was extended by a “control” scale. The already existing scale of dominance ranges from inferior to superior. The term “control” is supplemented in the questionnaire by the words “control of the situation.”

In the knowledge inquiry, the subjects were asked about their knowledge of the trigger words or gestures acquired in the interaction and the corresponding reactions of the robots (Figure 3, see Block 2 T2 and T4).

In the adapted Post-Study System Usability Questionnaire (7-point scale, 1 to 6 and “specification not possible”) (Lewis, 1992, 2002; Sauro and Lewis, 2012) and the adapted Godspeed questionnaire (question pairs) (Bartneck et al., 2009) the interaction tasks and the robots were evaluated (see Block 3 in Figure 3).

The post–post study questionnaire was used to find out which interactions were perceived as more pleasant and how subjects define frustration since several different emotions might relate individually to this emotion (such as hate, sadness, and others).

#### Protocol of the Wizard-of-Oz

The Wizard-of-Oz (WoO) indicated before the start of the experiment whether the participant was in the FRUST- or NOFRUST-group stating accordingly in the program of the robot: Should the subject be frustrated? Yes (key “y”) or no (key “n”). This selected the appropriate program in which it was already determined in which trial which errors would occur. So the errors were not selected during the interaction task by WoO, they were already predefined.

The robot “waked up” and moved to the initial position. This movement was not seen by the participants. The WoO saw the interaction task with the help of a camera placed above the participant and the robot.

The action of the WoO within a trial could be divided in three phases:

(1) Activation of the movement to the handover position
(2) Action after an error or no error answering the question, if the participant choose the right trigger (gesture or trigger)
(2a) in case of an error: the answer was “no.” A new ball transfer was possible. It started again with phase 1.
(2b) in case of no error: the answer was “yes” after choosing the right trigger and “no” after choosing the wrong trigger.
(3) Transfer the ball to the corresponding container after the right trigger. After the release of the ball, the next trial started with phase 1.

In the following the phases were explained in more detail:

(1) The movement to the handover position to pick up the ball was activated by pressing the button “t” on the keyboard after the participant called over the robot. Either the error occurred, or the handover succeeded. The robot moved to the waiting position.
(2a) In case of an error, the wizard indicated that the input of the trigger was wrong. The participant could call over again. The wizard answered the next question: Did the participant ask for a new ball transfer? y/n. The trial started again with phase 1.
(2b) In case of no error, the wizard indicated whether the input of the trigger (gesture or word or combination) was correct or incorrect: Was the input correct? y/n.
(2b1) if yes, the LED lighted green. The wizard answered to the next question for the direction of the ball release into the corresponding basket. The gripper released the ball according into the container on the right or left side of the robot.
(2b2) if no, the LED lighted red after answering the next question with “no” by the wizard: “Did the participant ask for a new ball transfer?” So the participant could test another trigger/trigger combination until the answer to the trigger choice question was “yes.” Than the gripper released the ball into the corresponding container on the right or left side of the robot.
(3) After the right trigger choice and the releasing of the ball the robot moved back to the starting position. The next trial started with phase 1.

#### Ethics Approval Statement

The experiment received a positive ethical vote from the ethics committee of the Technical University of Berlin.

#### Data Analysis

The statistical analyses were carried out using SPSS 22 IBM Corp. (2013). For analysis and in order to provide a clearer understanding of how reliable and “stable” the results are, 95% confidence interval (CI), effect size (ES) *r* (Cohen, 1988), and *p*-values were determined (Cumming, 2014). Small effect is *r* = 0.1, medium effect is *r* = 0.3, and large effect is *r* = 0.5 (Cohen, 1988; Gignac and Szodorai, 2016). Self-performance is a score of the NASA-TLX scale. *T*-tests and bivariate correlations were conducted.

The condition (FRUST or NOFRUST) is the independent variable.

## Results

The results are presented in several sections. The first section deals with the detectability of frustration and the definition of the term. Then the results about the behavioral reactions after the technical execution error in the video data follows. Finally, the influence of frustration on interaction factors and the evaluation of interaction and robot systems is presented. More details about the results are shown in tables (Tables 1–5). Each table contains columns of the time points and the factors that were considered, of the confidence intervals (lower and upper bound), the effect size *r* and the *p*-values.

**TABLE 1:** The table shows the results of the frustration scales of the NASA-TLX and the EaCQ (see also section “Frustration can be determined with subjective methods”).

Time point	Factors	Upper bound	Lower bound	Effect size	*p*-value
After first interaction	Frustration (EaCQ 1)	0.04	1.68	0.383	0.04
	Frustration (NASA 1)	14.84	49.52	0.616	0.001
After second interaction	Frustration (EaCQ 2)	0.43	1.76	0.541	0.002
	Frustration (NASA 2)	16.48	48	0.661	0.0003

**TABLE 2:** The table shows the results of the reaction of the participants after an error of the robot (see also section “Specific reactions after an error by the robot”).

Time point	Factors	Upper bound	Lower bound	Effect size	*p*-value
First interaction	Smile	0.13	0.82	0.482	0.008
	Laugh	0.17	0.82	0.507	0.004
	Facial expression overall	0.89	2.34	0.655	0.0001
Second interaction	Lick one’s lips	0.01	0.46	0.485	0.041
	Laugh	0.10	0.76	0.454	0.012
	Facial expression overall	0.61	2.47	0.548	0.002
FRUST first interaction	Body overall	–1.82	–0.43	0.822	0.007
FRUST second interaction	Lick one’s lips	–0.85	–0.04	0.667	0.035
	Cock one’s head	–0.85	–0.04	0.667	0.035

**TABLE 3A:** The table shows the results of the interaction factors scales of the SAM, the EaCQ, and the NASA-TLX (see also section “Dominance and sense of control differs between condition groups”).

Time point	Factors	Upper bound	Lower bound	Effect size	*p*-value
After first interaction (T2)	Control (SAM)	–1.84	–0.15	0.444	0.023
After second interaction (T4)	Control (SAM)	–2. 29	–0.74	0.622	0.0005
Change during first interaction	Dominance1 (SAM)	–1.4	–0.12	0.438	0.022
Change during first interaction	Dominance2 (SAM)	–1.35	–0. 17	0.490	0.014
	Control2 (SAM)	–2.39	–0.37	0.495	0.009

**TABLE 3B:** The table shows the correlation between frustration and interaction factors (see also section “Frustration correlated negative with dominance, control and self-confidence”).

Time point	Factors	Upper bound	Lower bound	Effect size	*p*-value
After first interaction (T2)	Frustration and arousal	0.295	0.789	0.578	0.001
	Frustration and dominance	–0.685	–0.128	–0.459	0.011
	Frustration and control	–0.779	–0.410	–0.601	0.0005
	Frustration and self-confidence	-0.754	-0.322	-0.576	0.001
	Frustration and eye-rolling	0.212	0.611	0.371	0.044
	Frustration and facial expression overall	0.157	0.707	0.476	0.008
	Frustration and mouth twisting	0.025	0.692	0.4	0.028
After second interaction (T4)	Frustration and arousal	0.140	0.842	0.562	0.001
	Frustration and dominance	–0.690	–0.076	–0.445	0.014
	Frustration and control	–0.842	–0.440	–0.673	0.000047
	Frustration and self-confidence	–0.858	–0.530	–0.717	0.000008
	Frustration and self-reported task performance	–0.844	–0.228	–0.587	0.001
	Frustration and head-shaking	–0.021	0.788	0.462	0.01
	Frustration and lips linking	–0.007	0.705	0.412	0.024
	Frustration and eyebrow pull together	–0.008	0.146	0.502	0.006
	Frustration and facial expression overall	0.010	0.125	0.451	0.005
	Frustration and breathing out	0.0002	0.131	0.490	0.012

**TABLE 4:** The table shows the results of the reaction of the participants after an error of the robot for both robots in comparison between the condition groups (see also section “Specific reactions after an error by the robot”).

		*Pepper*				*Panda*			
Time point	Factors	Upper bound	Lower bound	Effect size	*p*-value	Upper bound	Lower bound	Effect size	*p*-value
First interaction	Laugh	0.003	1.11	0.641	0.049				
	Smile					0.098	1.08	0.627	0.023
	Facial expression overall					1.24	3.73	0.807	0.001
Second interaction	Laugh	0.19	1.06	0.791	0.011				
	Facial expression overall	0.34	2.38	0.626	0.013				
	Speech overall	0.01	1.49	0.671	0.048				
	Lick one’s lips					0.039	0–85	0.667	0.035

**TABLE 5:** The table shows the results of the robot rating for both robots in comparison between the condition groups for each interaction (see also section “Robot Rating: Robots were evaluated different in condition groups”).

		*Pepper*				*Panda*			
Time point	Factors	Upper bound	Lower bound	Effect size	*p*-value	Upper bound	Lower bound	Effect size	*p*-value
First interaction	Easy to use	–3.03	–0.74	0.703	0.003	–2.48	–0.31	0.712	0.018
	Correction of errors	–3.49	–0.95	0.726	0.002				
	Easy to brief	–2.86	–0.36	0.622	0.016				
	Good task					–2.87	–0.13	0.636	0.036
	Pleasant use					–2.19	–0.028	0.524	0.045
	productivity					–2.34	–0.41	0.649	0.009
	Clarity of reactions					–2.97	–0.28	0.611	0.022
Second interaction	Easy to use					–3.67	–1.66	0.863	0.000099
	Good task	–2.14	–0.18	0.583	0.024	–3.31	–0.57	0.684	0.010
	Pleasant use	–2.02	–0.63	0.755	0.001	–3.38	–0.74	0.738	0.006
	Productivity	–2.43	–0.21	0.652	0.025	–4.03	–0.63	0.718	0.013
	Satisfaction	–2.46	–0.04	0.527	0.044	–3.49	–0.39	0.749	0.021
	Clarity of reaction	–2.94	–0.16	0.557	0.031				
	Easy to brief	–2.24	–0.04	0.529	0.043	–3.01	–1.10	0.802	0.000499
	learning to use					–2.87	–0.47	0.687	0.011
	Overall evaluation					–4.32	–0.24	0.729	0.034

Square brackets in the text signal a 95% CI, lower and upper bound. The effect size is *r*.

Before (T1 and T3) and after (T2 and T4) the respective interaction task with the robot, the participants completed questionnaires (SAM, EaCQ, and NASA-TLX) about their own perception (see also Figure 3, Block 2).

### Frustration Can Be Determined With Subjective Methods

**FIGURE 4:**
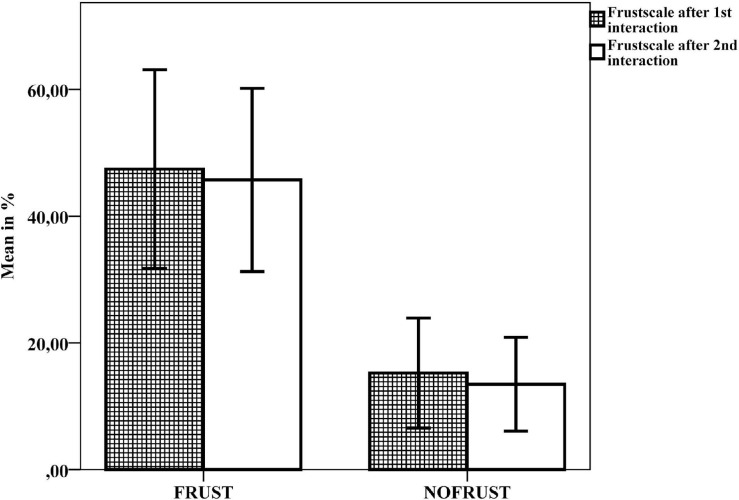
Results of the frustration scales of the NASA-TLX questionnaire after the first (T2) and second (T4) interaction task, mean values and 95% confidence intervals.

The frustration score of both questionnaires (NASA-TLX and EaCQ) was higher in the FRUST-group than in the NOFRUST-group after both interaction tasks (T2 and T4) (EaCQ 1: *MD* = 0.86 [0.04, 1.68], *r* = 0.383; NASA 1: *MD* = 32.18 [14.84, 49.52], *r* = 0.616; EaCQ 2: *MD* = 1.1 [0.43, 1.76], *r* = 0.541; NASA 2: *MD* = 32.24 [16.48, 48], r = 0.661) (Figure 4 and for more details see Table 1).

Participants were statistically not significant more frustrated in the interaction tasks with the robot “*Panda*” than with the robot “*Pepper*” (first interaction task: *MD* = 0.941 [–22.78, 21.97], *r* = 0,007; second interaction task: *MD* = –6.27 [–27.3, 14.77], *r* = 0,125). Moreover, there is no statistically significant difference whether participants first interacted with “*Pepper*” or with “*Panda*” in both conditions (first interaction: FRUST: *MD* = –6.54 [–38.42, 25.34], *r* = 0.115; NOFRUST: *MD* = 2.67 [–17.65, 22.98], *r* = 0.111; second interaction: FRUST: *MD* = –15.5 [–43.92, 12.91], *r* = 0.291; NOFRUST: *MD* = 10.76 [–2.86, 24.85], *r* = 0.516).

### Understanding of Term Frustration by Participants

In a free text field, the participants described what they understood by the word “frustration.” The participants also indicated which terms (terms from the NASA-TLX and which they themselves specified) they associate to what percentage with the term frustration. In the free definitions, the participants mainly indicated “disappointed expectations” and “not reaching a goal despite repeated attempts.” The term “annoyance” was given a high percentage, followed by “stress.” “Irritation” and “discouragement” were in average associated to frustration to more than 50%. Other terms frequently mentioned by the participants were “helplessness” and “disappointment.”

### Specific Reactions After an Error by the Robot

The videos of the HRIs were scanned for reactions of the participants to the errors of the robots. Then the frequencies of the reactions were counted, i.e., it was looked whether the reaction occurred at all in the interaction task and not how often in an interaction task. In addition, the reactions were summarized in four parameter groups: gestures, facial expressions, speech and body.

The results show that mainly facial expression are shown and in this parameter group, surprisingly, mainly laughter and smiles were found. There are mainly differences in the condition groups for these reactions (for more details see Tables 2, 4). Smiling and laughing was a frequent reaction after the occurrence of an error especially in the FRUST-group (see Table 2).

Frustration correlated positively with various reactions that participants exhibited following the robot’s errors in both interaction tasks (see also Tables 3A,B). In the first interaction task, there were positive correlations between frustration and facial expressions (*r* = 0.476 [0.157, 0.707]), such as eye-rolling (*r* = 0.371 [0.212, 0.611]) and mouth-twisting (*r* = 0.4 [0.025, 0.692]). In the second interaction task, there were also positive correlations between facial reactions (*r* = 0.502 [0.175, 0.737]), such as licking lips or pulling eyebrows together (*r* = 0.490 [0.232, 0.734]) and frustration. In addition, there were positive correlations between frustration and head shaking and breathing out.

### Dominance and Sense of Control Differs Between Condition Groups

Differences in control perception (SAM) between condition groups after the 1st (T2) and 2nd (T4) interaction task were found (SAM T2: *MD* = –0.995 [–1.84, –0.15], *r* = 0.444; SAM T4: *MD* = –1.51 [–2.29, –0.74], *r* = 0.622) (for more details see Table 3A).

There were differences between the groups for the factors of the SAM questionnaire items dominance and control before (T1 and T3) and after (T2 and T4) an interaction task (dominance T2: *MD* = –0.77 [–1.4, –0.12] *r* = 0.438; dominanceT4: *MD* = –0.76 [–1.35, –0.17], *r* = 0.490; control T4: *MD* = –1.38 [–2.39, –0.37], *r* = 0.495) (for more details see Table 3B).

### Frustration Correlated Negative With Dominance, Control and Self-Confidence

After the first (Block 2, T2) as well as the second (Block 2, T4) interaction task with the two robots a positive correlation between frustration and arousal was found (T2: [0.295, 0.789], *r* = 0.578; T4: [0.140, 0.842], *r* = 0.562). The correlations between frustration and the (respective) parameters dominance, control, self-confidence, and self-reported task performance are negative after both interaction tasks. The higher the frustration score, the lower the dominance score, the sense of control and self-report task performance (T2: dominance: [–0.685, –0.128], *r* = –0.459; control: [–0.779, –0.410], *r* = –0.601; self-confident: [–0.754, –0.322], *r* = –0.576; T4: dominance: [–0.690, –0.076], *r* = –0.445; control: [–0.842, –0.440], *r* = –0.673; self-confident: [–0.858, –0.530], *r* = –0.717). The subjects rated their task performance worse when frustration was high (T4: [–0.844, –0.228], *r* = –0.587) (for more details see also Table 3B).

### Robot Rating: Robots Were Evaluated Different in Condition Groups

**FIGURE 5:**
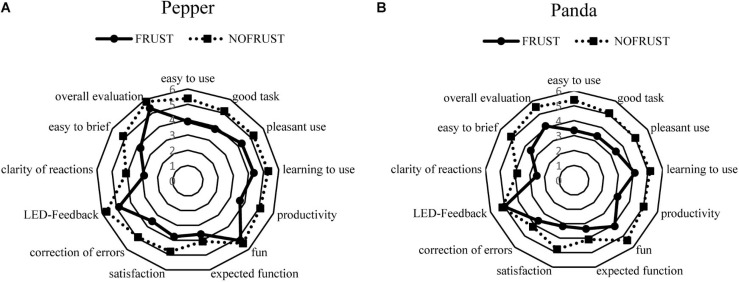
Evaluation of the robot systems [“*Pepper*” **(A)** And “*Panda*” **(B)**] independent of the sequence of interaction task.

After each interaction task both the robot and the interaction were evaluated with the Post-Study System Usability Questionnaire (Figure 3: Block 2, T2 and T4). Figure 5 shows the evaluation of each robot (“*Pepper*” and “*Panda*”) independent of the interaction sequence.

Both robot systems were rated better in the NOFRUST-group than in the FRUST-group independent of the sequence of interaction task (Figures 5A,B and see also Table 5). In the NOFRUST-group the robots were evaluated very similarly except for the category “correction of errors.” In the FRUST-group the robot “*Panda*” was rated worse than “*Pepper*,” except for the category “satisfaction” and “LED-feedback.”

**FIGURE 6:**
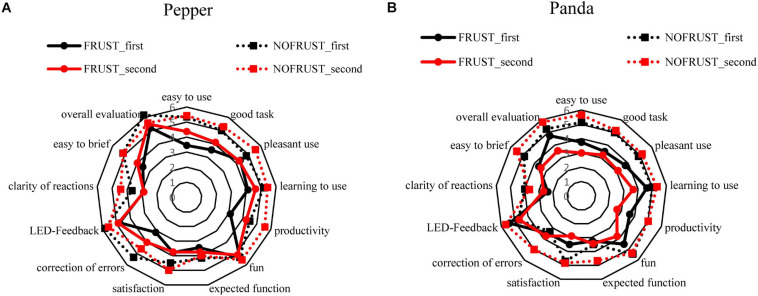
Evaluation of the robot systems [“*Pepper*” **(A)** And “*Panda*” **(B)**] after the first and second interaction task.

Participants in the NOFRUST-group described “*Pepper*” as more manageable and found it easier to correct its errors in both interaction tasks compared to the FRUST-group. In addition, the participants found “*Pepper*” easier to brief than in the FRUST-group (Figure 6A). Participants found the interaction task with “*Panda*” more productive and the robot easier to use in the NOFRUST-group than in the FRUST-group (Figure 6B). “*Panda*” was rated worse in more categories in the FRUST-group than in the NOFRUST-group and then “*Pepper*” in the FRUST-group (Figures 6A,B and see also Table 5).

There was no significant difference between the two robots in the FRUST-group on the indication of frustration and overall perception after the 1st interaction task (frustration: *MD* = –6.54 [–38.42, 25.34], *r* = 0.115; overall perception: *MD* = 0.47 [–0.67, 1.62], *r* = 0.224).

The robot “*Panda*” was rated more negatively than “*Pepper*” in the second interaction in the FRUST group in the following categories: easy to use (*MD* = 1.38 [0.06, 2.7], *r* = 0.506), pleasant use (*MD* = 1.14 [0.21, 2.06], *r* = 0.576), productivity (*MD* = 1.58 [0.28, 2.89], *r* = 0.576), fun (*MD* = 1.35 [0.42, 2.27], *r* = 0.655), overall perception (*MD* = 1.94 [0.89, 3.1], *r* = 0.726).

After the two interaction tasks, participants indicated which robot they preferred and why. “*Pepper*” was described as more human-like. It was attributed to be more trustworthy and “enabled more familiar interactions” with a more pleasant feeling. About “*Panda”* they stated that it was more functional, it was limited to the bare minimum of functionality, and the behavior was more expectable. More subjects preferred to interact with “*Pepper.*”

## Discussion

In this paper, results on the influence of frustration in a HRI study were presented and are discussed in the following section about recommendations for successful HRI.

### Short Description of the Study Design

In the reported study, participants performed a task in collaboration with a robotic system and with a common goal in a handover scenario. The participants interacted with two different robot systems, one after the other. There were two condition groups, frustration (FRUST) and no frustration (NOFRUST). Frustration was successfully induced through technical errors.

### Summary of the Results

In this section, the results are considered in relation to the hypotheses (see section “Hypotheses”).

#### H1: Technical errors by the robot lead to perceived frustration by the participants

The results showed that frustration occurred in the FRUST-group in both interaction tasks (with both robots). The operationalization of frustration was successful, also seen in the questionnaires (NASA-TLX and EaCQ). In the videos, reactions were found mainly in the faces of the participants, especially laughter and smiling. This is also reflected in statistical differences in the condition groups.

Participants defined frustration remarkably similar. Frustration is mainly associated with “disappointment,” especially with “expectations,” and “not reaching a goal.” These terms also correspond to the definition seen in several definitions in the literature in the introduction section (e.g., Freud, 1921; Russell, 1980; Amsel, 1992; Bortz and Doering, 2013).

Since facial expressions were very often shown in association with frustration, these might be good candidates to detect frustration in interaction situations. Usually specific facial expressions are expected, e.g., indicating frustration (Jost, 1941; Scherer, 2005; Hamm et al., 2011; Hazlett, 2013; Gao et al., 2014; Lerner et al., 2015). Therefore, a more careful way of detecting emotions is necessary, including the situational context. Detecting smiles and laughter by emotion detectors will not reflect the entire situation if the context of reoccurring failure is not considered.

#### H2: Frustration leads to decreased dominance, sense of control, and self-reported performance

Frustration affects dominance, the sense of control and self-confidence in both interaction situations. Frustration has shown negative correlation with all three characteristics.

As shown in other studies, frustration has an influence on interaction factors. We find the sense of dominance and control in interaction particularly relevant, which is very important for the evaluation of system and interaction quality and thus for a good collaboration. To be able to assess the situation is important for joint task accomplishment and collaboration as was mentioned earlier.

#### H3: Frustration leads to lower rating regarding acceptance of the robot systems

##### H4: The Interaction With the More Human-like Robot (Here “*Pepper*”) is Preferred, Among Other Aspects Due to the Human-like Appearance and Similarity to HHI

The robots have been evaluated differently in the condition groups, especially in the categories “easy to use,” “productivity,” and “easy to brief.” In the FRUST-group the robots were rated more negatively than in the NOFRUST-group. The robot “*Pepper*” was rated more positively on average than the robot arm “*Panda.*”

Frustration has an impact on the evaluation of interaction and robot systems. The experience of frustration seems to have a negative impact on the evaluation of the easy handling and the possibility to give good instructions. Thus, the task cannot be fulfilled as expected which in turn leads to disappointed expectations and frustration.

No significant difference was found between the frustration levels in the interaction tasks with the two robotic systems. Thus, the interaction task with the robot seemed to be independent in respect to the order of which robot is used first.

### What Are Relevant Factors for a Successful Human–Robot Interaction?

The following will describe the aspects which were examined in this study, how the results can be interpreted, and what this could mean for future HRI research.

#### Appearance

The two systems in this study differed in their appearance to examine if the appearance has an influence on the interaction. Furthermore, movement could form different expectations and might have a strong influence on the interactive behavior of the participant and on the evaluation of the interaction task.

The participants had more confidence in familiar situations and found the interaction with “*Pepper*” more natural and less disconcerting, probably because the robot looked more human-like and thus evoked the expectations of a HHI. This led to a better assessment of the reactions and movements, which in turn can increase the sense of dominance and control. Riek et al. (2009) found a positive effect of anthropomorphism, they showed that people empathize more with robots which have a more human-like than a mechanical appearance (Onnasch and Roesler, 2019) and treated them differently (Malle et al., 2016). But the robot appearance preferences depend on the environmental context (e.g., home versus factory) (Chanseau, 2019) and the task. The relevant issue is how good the evoked expectations through the appearance can be fulfilled through the robot in the specific task.

The appearance of the robot “*Panda*” was rated more negatively in several categories by the FRUST-group than “*Pepper*” in the second interaction task. Frustration seems to have a negative influence on the evaluation of the interaction and the interaction partner. Participants indicated that they found it easier to interact with “*Pepper,”* the interaction was more fun, and they found the robot to be better in the overall interaction rating. When indicating which robot the subjects preferred to interact with, the subjects indicated “*Pepper*” more often.

Expectations and attributions based on the appearance of the robot and the environment as well as the task have a great influence on the interaction, albeit mostly subconsciously. Therefore, attention should be paid to the associations that appearance and previously known abilities of the robot have on human partners. But not just the first impression is important also the performance of the robot influences subjective perception of the robot (Salem et al., 2015).

The appearance and capabilities of the robot system should be adapted to the scope of the interaction, for example, in certain areas it should be limited to the most necessary aspects and be more functional. In addition, the speed of the system in the interaction is important, whereby human safety must be guaranteed, but the interaction should be pleasant and (possibly) natural.

The robots were rated differently in the condition groups. Thus, perceived frustration had a negative impact on the rating of the interaction and the interaction partner. More participants indicated that they preferred interacting with “*Pepper*,” mainly because of appearance and familiarity. Appearance seems to be an important aspect in HRI. Thus, the study indicated that humans like to work with familiar objects and that the appearance of robotic systems should be suited to the context of use and functionally appropriate. Of course, the expectation triggered by the appearance should not be ignored.

#### Behavioral Reaction to Robots

The occurrence of the specific facial expressions, smiling and laughing, during the interaction tasks especially in the FRUST-group with both robots was an interesting aspect in this study. This was also reflected in the correlation results between frustration and behavioral data from the videos.

The ability to recognize facial expressions as additional information about human experience in interaction is an interesting aspect for the design of a robot system. The facial expressions, such as laughter and smiles, can be misinterpreted by the robot system if facial expressions are not interpreted in the situational context.

Furthermore, the ability to interpret emotional reactions correctly could be a valuable information in social robotic systems that make use of concepts like joint attention and common goal representation. This information gives a hint if the assumed common goal and necessary actions are aligned by both partners. This provides means to correct the assumed instances to come back to a successful collaborative interaction which would release the possible frustrating experience of the partner.

The participants showed two different types of reactions to the robot’s errors in interaction. The reactions were either directed toward the technical error or can be rated as attempts to correct the robot. Here, two types of errors can be differentiated, on the one hand traceable errors, which were more often treated with correction attempts, such as “hand-on-gestures” or color changes of the ball. On the other hand, non-traceable errors, whereupon only reactions, like facial expression, were shown. The error “gripper remains open” and “gripper remains closed” can be classified in the group of traceable errors, and the errors “accept ball and drop it in the human or robot area” are rather incomprehensible errors. Type of errors and intention of the robot influence anthrophomistic perception of the robot (Salem et al., 2015).

This shows the importance of research on cognitive modeling approaches that enable robots or intelligent systems to gain an understanding of the human partner (Kambhampati, 2019; Klaproth et al., 2020; Rußwinkel, 2020) in order to respond to the partner comprehensively. Furthermore, the robot needs to behave in a traceable fashion, so that the human partner is motivated to help even if errors occur. Just cases of pure “no comprehension” will be fatal for further interactions.

Thus, the study also showed that it is important to consider the behavior, especially the facial reactions of the human in the context of the interaction and that these are relevant for the course of the interaction. But without connecting the facial expression to the situation at hand, interpretations will remain difficult.

#### Dominance and Control

As shown in our previous study on frustration (Weidemann and Rußwinkel, 2019) the sense of dominance and control turn out to be important aspects in this context. The results revealed that frustration led to a reduction of sense of dominance and control.

The sense of dominance and control are important factors in an interaction and should be preserved for the human interaction partner (in the interaction). Negative emotions, such as discomfort, irritation, and frustration lead to the human partner to lose the sense of dominance and control which leads to a termination of the collaboration or at least to the negative evaluation of the interaction. Certainly, the acceptance of the robot system will decrease if the negative situation will not be solved.

Therefore, it is important to minimize negative emotions in the interaction. This can be achieved by for example fulfilling expectations, recognizing, and understanding human emotions and feelings, and showing the appropriate and desired feedback.

#### Feedback

An important aspect in the design of a good HRI is the feedback given by the robot to the human and also vice versa. For interpreting feedback reactions, it is important to understand if the partner has expected an event or agrees with the situation or decision of the partner.

For a good predictability, the robot should gather enough information about the human state and the human’s action to interpret this information in the appropriate context. With human partners, a major part of communication relies on the facial expression. Humans give immediate feedback to the robot in form of reactions such as facial expressions (Lang et al., 2010). Finding ways of interpreting such immediate facial expressions under consideration of the current situation is a promising approach for designing better collaborative robotic systems.

In case the feedback from the robot to the human, i.e., would be adapted to the human’s needs, would consider the situational context and would be accepted by the human, this would be considered as social feedback (Schneider, 2011). The feedback should serve the human being as support for the common fulfillment of the task, as well as representing the status and the next actions of the robot. This type of feedback can be realized through different channels, for example visual or haptic. LED feedback or other user interfaces are able to give immediate feedback.

The importance of interpreting and responding to facial expression was also demonstrated in this study. In addition, the use of LED-feedback helped in communication in fulfilling the common goal of the interaction task. Thus, the study showed that mutual feedback is important for pleasant HRI.

## Conclusion

In this study we were able to successfully induce frustration in a collaborative HRI situation by errors made by robots that lead to frustration by the human interaction partner and a delay in achieving the common goal. This way, we were able to validate the results of Giuliani et al. (2015) and Abd et al. (2017). The setup and protocol used for the study could be used in further studies that investigate measurements of frustration or means of reducing frustration, e.g., by a careful design of feedback signals or other kind. As we have argued, such situations and the impact on the human partner has a serious influence on successful HRI. In addition, the study provided indications about aspects that should carefully be considered in designing a good interaction with a robot. These aspects are robot appearance and feedback reactions the robot should provide to diminish frustration response by the human partner.

If these aspects are included in future HRIs, robots are more likely to be accepted in human life and in the working world and thus can lead to an “integration” of robots.

Frustration was determined in this study using questionnaires and behavioral reactions. To better identify frustration, we also included psychophysiological data (electrocardiogram, electrodermal activity, and electromyogram) in the study. These can be recorded in parallel with the occurrence of frustration. Alongside our behavioral data, this data will be investigated, analyzed and discussed in more detail in future work. It will be beneficial to gain a deeper understanding what circumstances lead to frustration – may be even how the feeling of agency or sense of control can be supported in interaction situations.

This may provide the robot with additional data about the human’s state during an interaction and allow it to recognize frustration or other emotions, and to respond appropriately. So they can help the human in his or her activities. Frustration can be minimized. How frustration can be minimized in a HRI should be investigated in future studies.

Another interesting question is whether and how the behavioral responses in the FRUST-group change over the interaction. Possibly, the changes of the frustration level could be determined by this data. This question should be investigated in another experimental design, also to measure frustration with different methods at multiple time points. Thus, parallel measurement to the occurrence of frustration with different methods and minimization of frustration should be investigated in future studies.

The fact that feedback between the interaction partners plays an important role was also made clear once again in this study. However, only the visual channel was considered in relation to the feedback by the robot (LED-Feedback). Which other or additional feedback channels are still suitable for HRI should be examined in further studies.

In general the study also provides evidence that it is of high relevance to consider emotional reactions in HRI which also provides information on the others expectations and motivations. This could be done by emotion recognition programs or by measuring arousal. But taken alone this will not help since the context of the situation the emotion changes is of high relevance for the interpretation. Emotional reactions therefore be considered as part of the communication that is taking part. Cognitive modeling could help to provide this kind of context as e.g., shown in neuro adaptive assistance systems or other approaches of human aware AI (Kambhampati, 2019).

In future studies the robot systems and interaction should be adapted according to the recommendations developed in this paper and be tested in interaction studies with similar tasks that take into account close interaction, feedback provided, evaluation of emotional reactions, behavioral data in non-functioning situations. Questions remain, how simple feedback reactions could lead to a better impression regarding sense of control. Or to find simpler methods to measure frustration and agency.

The main massage is, that more research is needed toward human aware robotic systems, modeling of the mental and cognitive state of the human partner for providing better anticipation skills, and to engage further into considering metrics for emotional reaction and interpretation.

## Data Availability Statement

The raw data supporting the conclusions of this article will be made available by the authors, without undue reservation.

## Ethics Statement

The studies involving human participants were reviewed and approved by Ethics Committee (EC) of the Institute for Psychology and Industrial Sciences (IPA), Technical University of Berlin. The patients/participants provided their written informed consent to participate in this study.

## Author Contributions

AW and NR designed the experiment, drafted, edited, revised, and approved the manuscript. AW performed the experiments and analyzed the data. Both authors contributed to the article and approved the submitted version.

## Conflict of Interest

The authors declare that the research was conducted in the absence of any commercial or financial relationships that could be construed as a potential conflict of interest.
